# Editorial: From bench to bedside, targeting secondary brain damage following intracerebral hemorrhage

**DOI:** 10.3389/fnins.2022.1021776

**Published:** 2022-10-17

**Authors:** Cheng Gao, Zhong Wang

**Affiliations:** ^1^Department of Neurosurgery, The First Affiliated Hospital of Harbin Medical University, Harbin, China; ^2^Soochow University, Suzhou, China

**Keywords:** intracerebral hemorrhage, secondary brain injury, inflammatory response, immune response, apoptosis

Intracerebral hemorrhage (ICH) is the most common form of hemorrhagic stroke, accounting for up to 15% of all strokes. ICH has greater morbidity and mortality than ischemic stroke (Krishnamurthi et al., 2013; Feigin et al., 2015). Although neurosurgeons have made great efforts to understand the pathophysiology of ICH, no evidence-based primary treatment exists. To date, therapeutic strategies have mainly targeted hematoma expansion and the resultant mass effect; however, trials of both surgical decompression of ICH *via* craniotomy (STICH) and minimally invasive surgery with thrombolysis in ICH evacuation (MISTIE) have shown no functional improvement compared with intensive medical management alone (Mendelow et al., 2013; Hanley et al., 2019).

Recently, a growing body of evidence has indicated that secondary brain damage may occur through pathological processes following ICH (Aronowski and Zhao, 2011; Loan et al., 2022), including thrombin cascade, inflammatory response, cell apoptosis and necrosis, and oxidative stress, that lead to irreversible neurovascular unit damage. Elucidating the underlying molecular and cellular pathophysiology may reveal novel therapeutic targets therein. This Research Topic on “Targeting secondary brain damage following intracerebral hemorrhage: from bench to bedside” comprises 8 articles that address different aspects of secondary brain damage after ICH onset, as well as putative agents targeting mechanisms of secondary brain damage that have been assessed in preclinical ICH models and clinical trials.

Patients with ICH with hematoma expansion often have worse outcomes; preventing early hematoma enlargement would potentially improve neurological outcomes. Tanaka and Toyoda reviewed the current methods used against early hematoma growth, such as meticulous blood pressure control, anticoagulant reversal, and hemostatic therapy. However, although these methods safely reduce hematoma expansion, they remain insufficient to translate into good outcomes.

To date, multiple translational ICH studies targeted at secondary brain damage have been assessed in preclinical murine ICH models. Zhang et al. performed transcriptome analysis in ICH rats that revealed upregulated neuroinflammation-associated genes. In particular, IFN-γ response genes were significantly activated, and anti-IFN-γ treatment reduced brain injury in ICH rats. Similarly, Wang et al. conducted multiples analysis in ICH mice treated with febuxostat and showed that febuxostat attenuated neurodegeneration and apoptosis through inhibition of the expression of inflammatory cytokines and improvements in blood-brain barrier integrity after ICH. In addition, dexmedetomidine has been reported to inhibit inflammatory cytokines and oxidative stress. An et al. showed that dexmedetomidine administration alleviated ICH-induced anxiety behaviors; this may be associated with modulation of TRPV4 channels in the astrocyte.

Neuroprotection of the peripheral injured brain tissue is a rational treatment approach to improve clinical outcomes of patients with ICH. Edaravone is a free radical scavenger with an antioxidant effect capable of reducing ICH-induced brain injury. Though edaravone has been adopted for use in acute ICH patients on the basis of a weak recommendation, studies of the effect of edaravone treatment on survival and long-term functional outcomes in patients with ICH have yielded mixed results. In a systematic review and meta-analysis of 38 randomized controlled trials that used edaravone in ICH patients, Qin et al. showed that edaravone is not associated with mortality reduction, and data on whether it improves long-term outcomes are insufficient.

Treatment in other forms of hemorrhagic stroke may shed light on improving ICH outcomes. Yu et al. summarized randomized controlled trials and prospective trials of drug interventions in patients with chronic subdural hematoma recurrence, concluding that atorvastatin, dexamethasone, and tranexamic acid had efficacy in improving recurrence. Among the drugs studied, atorvastatin combined with dexamethasone was strongly recommended. Tan et al. systematically reviewed 16 articles on treatment with resveratrol in animal models of preclinical subarachnoid hemorrhage and found that resveratrol treatment was able to improve outcomes in these models. Altogether, these studies suggest that these drugs may have potential application value in the treatment of ICH patients.

Jarrahi et al. proposed a method called remote ischemic conditioning that enhanced hematoma resolution and improved neurological outcomes *via* anti-inflammatory polarization in acute preclinical ICH models. This could be a promising noninvasive method for improving ICH patient outcomes.

Taken together, the articles in this Research Topic highlight the relevance of studying secondary brain injury following ICH. Notably, this compilation of articles provides a comprehensive overview of the mechanisms of secondary brain damage following ICH and potential treatments for it. Further research is needed to better understand the pathophysiology of secondary brain damage after ICH, and more rigorous randomized controlled trials should be conducted to validate clinical treatment.

## Author contributions

CG was responsible for the writing of the manuscript. ZW was responsible for the review and revision of the manuscript. Both authors contributed to the article and approved the submitted version.

## Conflict of interest

The authors declare that the research was conducted in the absence of any commercial or financial relationships that could be construed as a potential conflict of interest.

## Publisher's note

All claims expressed in this article are solely those of the authors and do not necessarily represent those of their affiliated organizations, or those of the publisher, the editors and the reviewers. Any product that may be evaluated in this article, or claim that may be made by its manufacturer, is not guaranteed or endorsed by the publisher.
